# Development of an Automobile Indoor Air Quality Grading Based on Acute and Chronic Risk Assessment

**DOI:** 10.3390/toxics13090754

**Published:** 2025-09-04

**Authors:** Ji-Yun Jung, Young-Hyun Kim, Eun-Ju Lim, Young-Jun Byun, Min-Kwang Kim, Hyun-Woo Lee, Cha-Ryung Kim, In-Ji Park, Ho-Hyun Kim, Cheol-Min Lee

**Affiliations:** 1 Department of Chemical and Environmental Engineering, Seokyeong University, Seoul 02713, Republic of Korea; jju1049@skuniv.ac.kr (J.-Y.J.); rladudgus128@skuniv.ac.kr (Y.-H.K.); ho04sh@skuniv.ac.kr (H.-H.K.); 2 Korea Testing Laboratory, Seoul 08389, Republic of Korea; limeju@ktl.re.kr (E.-J.L.); younghost@ktl.re.kr (Y.-J.B.); kmk9404@ktl.re.kr (M.-K.K.); 3 Korea Transportation Safety Authority, Hwaseong 18247, Republic of Korea; peterlee@kotsa.or.kr (H.-W.L.); cha1052@kotsa.or.kr (C.-R.K.); coolinji@kotsa.or.kr (I.-J.P.)

**Keywords:** automobile, indoor air quality, health risk assessment, IAQ Grading

## Abstract

This study aimed to quantitatively evaluate the potential health effects of exposure to major air pollutants inside newly manufactured automobiles and to develop a grading system for automobile indoor air quality based on this assessment. To achieve this, the concentrations of 28 air pollutants were measured in five different automobile models. Among these, 18 substances were selected for health risk assessment based on the availability of acute and chronic toxicity data and the requirement that each substance had been detected at least once under one or more of the automobile test modes (AM, PM, and DM). Acute hazard quotients (HQ_acute_), chronic non-carcinogenic hazard quotients (HQ), and excess lifetime cancer risks (ECR) were subsequently calculated. The results of acute and chronic health risk assessments showed significant variation depending on the automobile test mode, and some automobiles exceeded health-based reference values for certain pollutants. Based on these findings, this study developed a 10-level grading system for automobile indoor air quality by comprehensively integrating pollutant-specific health risk levels and exceedances of the recommended limits outlined in Ministry of Land, Infrastructure, and Transport’s “Indoor Air Quality Guidelines for Newly Manufactured Automobiles.” The grading scale ranges from Grade 1 (Excellent) to Grade 10 (Hazardous), reflecting both acute and chronic health risks as well as legal standards, thereby improving upon conventional concentration-based management approaches. The proposed grading system enables a quantitative interpretation of automobile indoor air quality from a health-based perspective and is expected to be applicable in various fields, including automobile manufacturers’ air quality control, consumer information disclosure, and policy development.

## 1. Introduction

It has been reported that modern individuals spend more than 80~90% of their time indoors, with Koreans spending as much as 95.4% of their time in indoor environments [1,2]. Automobiles, a primary mode of transportation in modern life, have become an essential part of daily mobility, and the time spent inside automobiles is estimated to account for approximately 3% of a day. Furthermore, the number of registered automobiles in Korea has been steadily increasing since 2015, reaching approximately 26.3 million. This corresponds to 2.02 automobiles per person based on the national population of 51,684,564 [3,4]. These figures suggest that, although the time spent inside an automobile may be relatively short, the number of individuals exposed to the indoor environment of automobiles is continuously increasing.

According to a research report by the Ecology Center in the United States, as many as 275 different chemical substances have been identified inside the cabin of an automobile [5]. Previous research indicates that the type of interior material significantly influences VOC emissions and associated health risks [6]. Factors influencing automobile indoor air quality include internal sources such as automobile paint and interior materials, as well as external sources such as automobile exhaust emissions and the surrounding road air environment [7]. Among these, the primary contributors are known to be interior components—such as seats, carpets, consoles, door trims, and instrument panels—which release various chemical substances. Representative compounds include Volatile Organic Compounds (VOCs) such as Benzene and Toluene, and carbonyl compounds such as Formaldehyde [8]. A comprehensive review has identified plastics, rubber, leather (synthetic and natural), fabrics, and adhesives as primary sources of in-cabin VOCs in new automobiles [9]. These VOCs, once emitted, can cause what is often referred to as “new car syndrome,” which is analogous to “Sick House Syndrome (SHS),” leading to symptoms such as headaches, irritation of the eyes and skin, and an unpleasant odor. Even at low concentrations, these substances can negatively affect human comfort and may be associated with various health issues [10,11].

In recent years, growing recognition of the need for integrated management of complex air pollutants—rather than individual pollutants—has led to the development and use of various air quality indices such as the Air Quality Index (AQI), Air Pollution Index (API), and Air Quality Health Index (AQHI) [12,13,14]. In Korea, the government has introduced the Comprehensive Air Quality Index (CAI) to communicate air pollution levels to the public. This index was designed to reflect both the health effects and the perceived severity of air pollution, serving as a graded classification system for ambient air quality [15]. However, indoor air quality grading in Korea has largely focused on building environments, and rather than being driven by government initiatives, existing grading systems have primarily been developed by sensor manufacturing companies as part of consumer services in response to the expanding indoor air quality monitoring market. In many cases, such grading systems remain at the proposal or experimental research stage [10]. In particular, the importance of managing the indoor environment of automobiles, the main means of transportation for modern people, is receiving attention, and while management methods such as automobile environmental performance evaluation programs are already being utilized in Europe and China, there is no development of an automobile indoor air quality grading system in Korea.

Therefore, this study aimed to derive an automobile indoor air quality grading system that considers the potential health impacts of pollutants absorbed by the human body, rather than the conventional concentration-based management method. Accordingly, we developed and presented a new automobile indoor air quality grading system that considers the potential for acute and chronic adverse health effects from short-term exposure to various hazardous air pollutants in new automobiles and long-term exposure over the automobile’s lifespan.

For clarity, all abbreviations and notations used throughout this study are summarized in the nomenclature list provided in the Supplementary Materials (Table S1).

## 2. Materials and Methods

### 2.1. Measurement and Analysis of Pollutants in Automobile Indoor Air

In this study, the automobile indoor air quality grading system developed using empirical data was validated through the measurement and analysis of air pollutants inside automobiles. Measurements were conducted in accordance with the “Indoor Air Quality Guidelines for Newly Manufactured Automobiles” issued by the Ministry of Land, Infrastructure, and Transport, as well as the “Standard Methods for Air Quality” and “Standard Methods for Indoor Air Quality” published by the National Institute of Environmental Research [16,17,18]. Five domestic and imported automobile models were tested under three operational modes—Ambient Mode (AM), Parking Mode (PM), and Driving Mode (DM)—to measure 28 target pollutants, including VOCs, carbonyl compounds, and ammonia. In the AM condition, automobiles were soaked for approximately 16 h at a chamber temperature of 25 °C and Relative Humidity (RH) of 50%, followed by sampling. In the DM condition, the engine and air conditioning system were operated under heating conditions using infrared radiation at 25 °C and 50% RH, and samples were collected immediately after activation. For PM, samples were taken after 2, 4, and 6 h of soaking under heated conditions at the same temperature and humidity.

VOCs were collected using an MP-∑30 KN air sampler (SIBATA Scientific Technology Ltd., Tokyo, Japan) with Tenax TA-packed adsorption tubes at a flow rate of 100 mL/min for approximately 20 min. The tubes were thermally desorbed and analyzed using Gas Chromatography with a Flame Ionization Detector (GC/FID). Carbonyl compounds were collected using DNPH-derivatized cartridges at a flow rate of 1 L/min for about 20 min. DNPH derivatives were extracted using Acetonitrile, and a portion of the solution was analyzed using High-Performance Liquid Chromatography (HPLC) with a UV detector set at 360 nm. Ammonia was analyzed using the Boric acid absorption method combined with UV/Vis spectrophotometry. Air was drawn into 0.5% Boric acid solution using an LV-40BR sampler (SIBATA Scientific Technology Ltd., Tokyo, Japan). For analysis, Phenol-nitroprusside and sodium hypochlorite solutions were added to the sample, and absorbance was measured at 640 nm using a spectrophotometer (Agilent Cary 3500, Agilent, Santa Clara, CA, USA).

Quality assurance and quality control (QA/QC) procedures were conducted to ensure the reliability of the measurement and analysis. The QA/QC results are summarized in Table 1. Instrumental reproducibility was assessed by repeatedly analyzing standard materials and confirming the consistency of Retention Times (RTs). The Method Detection Limit (MDL) for each target pollutant was determined by performing seven replicate measurements at concentrations near the expected MDL and then multiplying the Standard Deviation (SD) of the measured values by 3.14.

### 2.2. Development of an Automobile Indoor Air Quality Grading System

To develop an automobile indoor air quality grading system based on the potential health effects caused by exposure to various hazardous air pollutants in newly manufactured automobiles, it was deemed necessary to conduct both acute and chronic risk assessments. This need arises from the exposure characteristics of automobile users, the possibility of high concentrations of certain pollutants at the time of automobile delivery, and the consideration of long-term exposure over the automobile’s lifespan. In this study, a health-based grading method was proposed through acute and chronic risk assessments of exposure to hazardous pollutants in the indoor environment of newly manufactured automobiles. To develop an automobile indoor air quality grading system that reflects real-world exposure patterns of users, acute and chronic exposures were distinguished, and hazard quotients for each pollutant were calculated to determine the grade based on whether the threshold values were exceeded.

First, the Acute Hazard Quotient (HQ_acute_) was calculated by dividing the concentration of each pollutant in the automobile cabin by its corresponding Minimal Risk Level (MRL), as shown in Equation (1). To obtain information on the acute toxicity of hazardous substances, the presence of acute MRLs was verified based on the values provided by the Agency for Toxic Substances and Disease Registry (ATSDR). The MRL is a reference value used in public health that estimates the daily human exposure to a hazardous chemical that is unlikely to pose a noticeable risk of non-carcinogenic adverse effects over a specified exposure duration [19]. An HQ_acute_ exceeding 1 was considered indicative of a potential risk of acute health effects.

(1) $${HQ}_{acute}=\frac{C}{MRL}$$

Here, C refers to the concentration of each pollutant (μg/m^3^), and MRL stands for Minimal Risk Level (μg/m^3^). The calculated HQ_acute_ was then evaluated to determine whether it exceeded the threshold value of 1.

For the chronic risk assessment, the health risk assessment (HRA) methodology established by the United States Environmental Protection Agency (US EPA) was applied. Information on the chronic toxicity of hazardous pollutants was collected using the US EPA’s Integrated Risk Information System (IRIS), including each pollutant’s inhalation-based Unit Risk (UR) for carcinogens and Reference Concentration (RfC) for non-carcinogens. The UR for carcinogens was converted to a Cancer Potency Factor (CPF) by incorporating standard body weight and inhalation rate, while the RfC for non-carcinogens was converted to a Reference Dose (RfD) using the same approach (Equations (2) and (3)). The body weight and inhalation rate values used in these conversions were based on US EPA standards for adults: 70 kg for body weight and 20 m^3^/day for inhalation rate [20].

(2) $$CPF[{\left(\mu g/kg/day\right)}^{-1}]=\frac{UR\left[{\left(\mu g/m^{3}\right)}^{-1}\right]\times 70\;kg}{20\;m^{3}/day}$$

(3) $$RfD(\mu g/kg/day)=\frac{RfC\left(mg/m^{3}\right)\times 20\;m^{3}/day}{70\;kg}\times 1000\;\mu g/mg$$

To reflect the actual exposure characteristics of automobile users, exposure factors used in the exposure assessment were derived from a user survey that investigated the primary duration, frequency, and patterns of automobile use. Based on the survey results, Exposure Time (ET) and Exposure Frequency (EF) were calculated and applied. Inhalation Rate (IR) and Body Weight (BW) were obtained from the “Exposure Factors Handbook for the Korean Population” published by the Ministry of Environment [3]. Exposure Duration (ED) was set to 15 years, based on the average automobile scrappage cycle reported by the Korea Auto Dismantlement Recycling Association [21]. This duration was converted to days and used as the Average Time (AT) for non-carcinogenic risk assessment. Lifetime (LT) was set to 83 years, following the 2023 national life expectancy reported by Statistics Korea, and was also converted to days for application [22]. A summary of the exposure factors used in this study is provided in Table 2.

For carcinogens, the Excess Cancer Risk (ECR) was calculated by multiplying the Lifetime Average Daily Dose (LADD) by the CPF derived through dose–response assessment. For non-carcinogens, the Hazard Quotient (HQ) was determined by dividing the Average Daily Dose (ADD), representing the estimated exposure over a certain period, by the RfD obtained through dose–response assessment [20]. The equations used to calculate ECR and HQ are presented in Equations (4) and (5).

(4) $$ECR=CPF\times LADD=CPF\times \frac{C\times IR\times ET\times EF\times ED}{BW\times LT}$$

(5) $$HQ=\frac{ADD}{RfD}=\frac{\frac{C\times IR\times ET\times EF\times ED}{BW\times AT}}{RfD}$$

Here, C refers to the concentration of each pollutant (μg/m^3^), IR is the Inhalation Rate (m^3^/day), ET is the Exposure Time, EF is the Exposure Frequency (days/yr), ED is the Exposure Duration (yr), BW is the Body Weight (kg), LT is the Lifetime (days), AT is the Average Time (days), CPF is the Cancer Potency Factor [(μg/kg/day)^−1^], and RfD is the Reference Dose (μg/kg/day).

For carcinogens, the calculated ECR was evaluated against thresholds of 1 × 10^−6^, 1 × 10^−5^, and 1 × 10^−4^. For non-carcinogens, the calculated HQ was compared to thresholds of 1 and the more conservative value of 0.1.

In this study, the automobile indoor air quality was classified into ten grades based on whether the thresholds for acute risk, chronic carcinogenic risk, and chronic non-carcinogenic risk were exceeded. The grading scale ranged from Grade 1 to Grade 10, defined as “Excellent”, “Very Good”, “Good”, “Moderate (High)”, “Moderate”, “Moderate (Low)”, “Slightly Unhealthy”, “Unhealthy”, “Very Unhealthy”, and “Hazardous”. Pollutant concentrations measured in five different automobiles under each test mode were applied to the grading methodology developed in this study to determine the air quality grade for each automobile and test condition. This process was conducted to evaluate the applicability of the proposed automobile indoor air quality grading system.

## 3. Results and Discussion

### 3.1. Development of an Indoor Air Quality Grading System for Automobiles

In this study, the grading system was developed based on acute and chronic health risk assessments; therefore, only air pollutants with available toxicological data for both acute and chronic inhalation exposure were selected. Accordingly, the final list of pollutants used for automobile indoor air quality grading was determined by selecting substances that were detected at least once in the indoor air monitoring results from newly manufactured automobiles under each test mode, and for which both acute and chronic toxicity data were available, while excluding those with values reported as not detected. In addition, eight substances—Formaldehyde, Benzene, Toluene, Xylene, Ethylbenzene, Styrene, Acrolein, and Acetaldehyde—were included in the list of target pollutants, as they are currently subject to recommended limits under the Ministry of Land, Infrastructure, and Transport’s “Indoor Air Quality Guidelines for Newly Manufactured Automobiles” in Korea. A total of 18 air pollutants were ultimately selected for use in the development of the automobile indoor air quality grading system. These pollutants include Acrylonitrile, Methylene Chloride, 1,2-Dichloroethane, Benzene, Toluene, Ethylbenzene, m-Xylene, p-Xylene, Styrene, o-Xylene, 1,2,4-Trimethylbenzene, Methyl Ethyl Ketone, Hexane, Formaldehyde, Acetaldehyde, Acrolein, Propionaldehyde, and Ammonia. The acute and chronic toxicity values, as well as the concentrations of each pollutant measured under different automobile test modes, are presented in Table 3 and Table 4.

For acute risk, the grading criteria were based on whether the acute reference value was exceeded, with an HQ_acute_ of 1 set as the threshold. For chronic risk, carcinogenic substances were evaluated using thresholds of 1 × 10^−^^6^, 1 × 10^−^^5^, and 1 × 10^−^^4^, in reference to the acceptable risk range proposed by the US EPA [44]. For non-carcinogenic substances, the thresholds were set at 1 (the standard acceptable level) and 0.1 (a more conservative value, ten times stricter). However, since the Ministry of Land, Infrastructure, and Transport [16] provides recommended concentration limits for eight pollutants—Formaldehyde, Benzene, Toluene, Xylene, Ethylbenzene, Styrene, Acrolein, and Acetaldehyde—in the “Indoor Air Quality Guidelines for Newly Manufactured Automobiles,” these regulatory standards were given priority in the grading process. Specifically, if the concentration of any of these eight substances exceeded its respective recommended limit, the automobile was assigned the lowest grade (Grade 10), even if the health risk thresholds were not exceeded. The final grading system incorporating both acute and chronic risk assessments is presented in Table 5.

In comparing the grading system developed in this study with international standards, several points of alignment and distinction can be noted. In the European Union (EU), for example, the REACH Regulation 2023/1464 has recently established a Formaldehyde emission limit of 0.062 mg/m3 for automobile interiors, which will become enforceable in 2027 [45]. This highlights the growing importance of regulating in-cabin pollutants at the policy level. Similarly, Japan has taken an early initiative through the Japan Automobile Manufacturers Association (JAMA), which issued voluntary guidelines in 2005 to reduce VOC concentrations in automobile cabins, supported by standardized testing protocols such as JASO Z 125 [46]. By contrast, the United States has not yet implemented enforceable federal standards specific to automobile indoor air quality, although agencies such as the California Air Resources Board (CARB) and US EPA have developed VOC definitions and guidelines that indirectly influence the sector [47,48].

These comparisons suggest that the grading system proposed in this study is not only consistent with the direction of international regulatory trends but also adds value by integrating both acute and chronic health risk thresholds. In particular, the incorporation of an exception rule based on national guideline values ensures compatibility with existing policies, while the health-based framework strengthens its scientific foundation. Taken together, these features indicate that the grading system could serve as a practical tool with relevance not only within Korea but also in broader global contexts.

### 3.2. Grading-Based Evaluation of Automobile Indoor Air Quality

#### 3.2.1. Acute and Chronic Risk Assessment for Automobile Users

In this study, acute, chronic non-carcinogenic, and chronic carcinogenic risk assessments were conducted for 18 air pollutants, and the corresponding HQ_acute_, HQ, and ECR values were calculated for each pollutant. The results of HQ_acute_, HQ, and ECR calculations for each automobile and each pollutant are summarized in Table 6, Table 7, Table 8, Table 9 and Table 10. For Automobile A, the results of the acute risk assessment indicated that no pollutant exceeded an HQ_acute_ of 1 under any test mode, suggesting that the potential for acute health effects due to short-term exposure was negligible. In the chronic non-carcinogenic risk assessment, at least one pollutant with an HQ exceeding 0.1 was identified in all test modes. In the chronic carcinogenic risk assessment, the ECR value for Acrylonitrile exceeded 1 × 10^−5^ under both the PM (4 h) and PM (6 h) conditions. For Automobiles B and D, the results of the acute risk assessment showed that the HQ_acute_ for Formaldehyde exceeded 1 under all PM conditions. In the chronic non-carcinogenic risk assessment, at least one pollutant with an HQ exceeding 0.1 was identified in all test modes. In the chronic carcinogenic risk assessment, the ECR for Acrylonitrile exceeded 1 × 10^−5^ in all PM conditions. For Automobile C, the results of the acute risk assessment indicated that no pollutant exceeded an HQ_acute_ of 1 under any test mode, suggesting no potential for acute health effects due to short-term exposure. In the chronic non-carcinogenic risk assessment, at least one pollutant with an HQ exceeding 0.1 was identified under AM and PM conditions. In the chronic carcinogenic risk assessment, at least one pollutant with an ECR exceeding 1 × 10^−6^ was identified under both AM and PM conditions. For Automobile C, the results of both acute and chronic risk assessments under the DM condition showed that no pollutant exceeded the relevant threshold values, indicating no potential health effects associated with exposure to indoor air pollutants during automobile operation. For Automobile E, the acute risk assessment revealed that at least one pollutant had an HQ_acute_ exceeding 1 under both PM and DM conditions. In the chronic carcinogenic risk assessment, the ECR for Acrylonitrile and Formaldehyde exceeded 1 × 10^−5^ under both the PM (4 h) and PM (6 h) conditions.

In most automobiles, the HQ_acute_ values did not exceed 1, indicating that acute exposure to pollutants is unlikely to pose health risks. However, under PM conditions—characterized by elevated temperatures and prolonged exposure—Formaldehyde exhibited HQ_acute_ values exceeding the threshold in some cases. This is likely due to increased emission rates from interior materials as the cabin temperature rises while the automobile is parked [49]. Similarly, in the chronic risk assessment, Formaldehyde was found to exceed threshold values, suggesting potential long-term health effects. Previous studies by Liang et al. [50] and Xu et al. [51] also reported ECR values exceeding 1 × 10^−6^, indicating potential carcinogenic risks associated with Formaldehyde exposure. Their findings further support the results of this study, particularly in showing high emission levels under high-temperature parking conditions. In addition, Acrylonitrile was found to exceed the chronic risk thresholds in this study. This result aligns with the findings of Jeon et al. [52], which reported that Acrylonitrile exhibited ECR values exceeding 1 × 10^−6^ in multiple automobiles.

Under PM conditions, the combination of high temperature and a sealed environment was found to significantly increase the cumulative emission of pollutants, frequently resulting in exceedances of both acute and chronic risk thresholds [49]. It was also observed that the longer the duration of PM conditions, the higher the level of health risk. Similarly, investigations of thermal comfort in extreme conditions and effects of prolonged mask wearing on perceived air quality indicate that thermal and micro-environmental factors can exacerbate exposure risks [9,53]. In contrast, under DM conditions, the HQ and ECR values for most pollutants tended to remain low. This is likely due to the operation of the automobile’s air conditioning and ventilation systems, which are effective in reducing the concentration of VOCs [9]. AM is a test condition that simulates pollutant emissions under environmental conditions similar to the outside atmosphere. Because this mode assumes non-operational, enclosed conditions without ventilation, it reflects potential exposure to accumulated pollutants. As a result, certain automobiles exhibited higher HQ and ECR values under AM than under DM [54]. Considering the specific characteristics of each test mode and the corresponding risk assessment results, it is suggested that further research be conducted on management strategies to improve automobile indoor air quality. These may include the development of VOC filtration technologies, automatic ventilation systems, temperature-activated window opening mechanisms, and the installation of in-cabin air purification devices [55].

#### 3.2.2. Application Results of Automobile Indoor Air Quality Grading

The results of applying the automobile indoor air quality grading system developed in this study based on acute and chronic risk assessment methodologies—to five automobile models—are summarized in Table 11. Acute and chronic health risk assessments were conducted using the pollutant concentration data measured from each automobile, and the HQ_acute_, HQ, and ECR values were evaluated against the respective threshold levels to determine the grading for each test mode. For Automobile A, no pollutant exceeded an HQ_acute_ of 1 under AM, DM, and PM (2 h) conditions. However, HQ exceeded 0.1 and ECR exceeded 1 × 10^−^^6^ but did not exceed 1 × 10^−^^5^, resulting in a final grade of 5. Under PM (4 h) and PM (6 h) conditions, HQ_acute_ also remained below 1, but HQ exceeded 0.1 and ECR exceeded 1 × 10^−^^5^, leading to a final grade of 7. For Automobiles B and D, HQ_acute_ values remained below 1 under both AM and DM conditions. However, HQ exceeded 0.1 and ECR exceeded 1 × 10^−^^6^ but not 1 × 10^−^^5^, resulting in a final grade of 5. In PM (2 h), PM (4 h), and PM (6 h) conditions, at least one pollutant exceeded an HQ_acute_ of 1. Additionally, HQ exceeded 0.1 and ECR exceeded 1 × 10^−^^5^, resulting in a final grade of 9. For Automobile C, no pollutant exceeded an HQ_acute_ of 1 under AM, PM (2 h), PM (4 h), or PM (6 h) conditions. However, HQ exceeded 0.1 and ECR exceeded 1 × 10^−^^6^ but did not exceed 1 × 10^−^^5^, resulting in a final grade of 5. Under DM conditions, HQ_acute_ remained below 1, HQ did not exceed 0.1, and ECR was also below 1 × 10^−^^6^, leading to the best possible grade, grade 1. For Automobile E, under AM conditions, no pollutant exceeded an HQ_acute_ of 1, and HQ remained below 1; however, ECR exceeded 1 × 10^−^^6^, resulting in a final grade 3. In PM (2 h), PM (4 h), and DM conditions, at least one pollutant exceeded an HQacute of 1. Additionally, HQ exceeded 0.1 and ECR exceeded 1 × 10^−^^5^, resulting in a final grade of 9. Under PM (6 h) conditions, the concentration of m-Xylene (943.6 μg/m^3^) exceeded the recommended limit for Xylene (870 μg/m^3^), as specified in the “Indoor Air Quality Guidelines for Newly Manufactured Automobiles” by the Ministry of Land, Infrastructure, and Transport. Therefore, in accordance with the exception rule defined in this study’s grading system, the automobile was assigned the lowest grade, grade 10.

While existing indoor air quality grading systems in Korea have primarily been designed for building environments, the grading system developed in this study is differentiated by its focus on automobiles and its incorporation of real-world exposure conditions experienced by users. In particular, by comprehensively considering both acute and chronic health risks, this system offers a health-based approach rather than a simple concentration-based one. Moreover, the system was designed to assign the lowest grade, grade 10, in cases where any of the eight pollutants regulated by the Ministry of Land, Infrastructure, and Transport exceeds its recommended limit, even if the calculated health risks are relatively low. This ensures that the official guideline values are reflected and enhances the system’s discriminatory power. This study is significant in that it presents the first automobile indoor air quality grading system in Korea based on both acute and chronic risk assessment, thereby expanding the potential for application in related policy domains. However, a key limitation is that the grading system was developed exclusively for newly manufactured automobiles and does not fully account for the variability across different automobile types or climatic conditions. Future research should address this by applying the grading system to a broader range of vehicle categories and incorporating real-world environmental factors to enhance its generalizability and practical utility.

## 4. Conclusions

This study established a ten-level automobile indoor air quality grading system derived from acute and chronic health risk assessments. While various air pollutants were detected in newly manufactured automobiles, the results indicated that substances such as Formaldehyde, Acrolein, and Acrylonitrile may exceed health-based thresholds under certain conditions, suggesting potential acute and health risks for automobile users. To address this, the grading framework was designed to integrate both risk assessment outcomes and recommended limits specified by the Ministry of Land, Infrastructure, and Transport, thereby moving beyond a simple concentration-based management approach. An exception rule was also incorporated, ensuring that any exceedance of the regulatory guideline automatically results in the lowest grade assignment.

The proposed grading system offers a practical and health-oriented evaluation tool that can be applied to automobile manufacturing, consumer information, and policy development. By quantitatively grading automobile indoor air quality from “Excellent” to “Hazardous,” this study provides the first health-based framework in Korea that considers both short-term and long-term exposure risks. These findings underscore the importance of systematic management of in-cabin air quality, and future studies should expand the application to a wider range of automobile models and real-world operating conditions to enhance the robustness and applicability of the system.

## Figures and Tables

**Table 1:** Quality assurance and quality control results.

Pollutants	Linearity	Detection Limit (μg/m^3^)	Quantification Limit (μg/m^3^)	Analysis Precision [RT]	Analysis Precision [Area]
Acrylonitrile	0.9986	3.4	11.0	0.002	2.7
Methylene Chloride	0.9999	6.4	20.4	0.001	3.9
1,2-Dichloroethane	1.0000	11.8	37.6	0.007	1.9
Benzene	0.9999	1.7	5.3	0.006	2.0
Toluene	0.9998	5.3	17.0	0.002	2.5
Tetrachloroethylene	1.0000	7.5	23.8	0.002	2.2
Ethylbenzene	0.9999	5.3	16.9	0.003	2.6
m-Xylene	1.0000	9.9	31.5	0.003	5.5
p-Xylene	0.9996	9.1	29.0	0.003	1.5
Styrene	0.9997	2.9	9.3	0.003	3.0
o-Xylene	1.0000	5.8	18.3	0.003	2.6
1,2,4-Trimethylbenzene	0.9987	2.5	8.0	0.001	3.2
Methyl Ethyl Ketone	0.9980	5.9	18.7	0.007	2.3
Iso-butanol	0.9995	6.7	21.5	0.007	1.9
n-Butyl Acetate	1.0000	6.2	19.9	0.003	2.2
Ethyl Acetate	0.9989	4.4	14.0	0.006	2.6
Hexane	0.9922	8.9	28.5	0.006	5.9
n-Butanol	0.9974	3.6	11.6	0.005	3.9
n-Decane	0.9998	3.7	11.8	0.001	1.2
Nonanal	0.9999	4.7	14.9	0.001	1.1
n-Undecane	0.9997	4.1	13.4	0.001	1.1
n-Dodecane	0.9951	3.8	12.2	0.001	1.2
Formaldehyde	1.0000	0.2	0.5	0.001	0.3
Acetaldehyde	1.0000	0.2	0.6	0.001	0.2
Acetone	1.0000	0.4	1.2	0.002	0.3
Acrolein	1.0000	0.3	0.9	0.002	0.3
Propionaldehyde	1.0000	0.4	1.3	0.003	0.6
Ammonia	0.9999	0.6	1.8	-	0.1

**Table 2:** Exposure factors for automobile users.

Exposure Factors		Values	Reference
Inhalation Rate (IR)	m^3^/day	14.62	[3]
Exposure Time (ET)	-	0.084	This study
Exposure Frequency (EF)	day/yr	275.6	
Exposure Duration (ED)	yr	15	[21]
Body Weight (BW)	kg	64.5	[3]
Average Time (AT)	day	5475	[21]
Lifetime (LT)	day	30,185	[22]

**Table 3:** Acute and chronic toxicity information by pollutant.

Pollutants	MRL ^a^ (μg/m^3^)	UR ^b^ [(μg/m^3^)]^−1^	CPF ^c^ [(μg/kg/day)^−1^]	RfC ^d^ (mg/m^3^)	RfD ^e^ (μg/kg/day)	MOLIT Standards ^f^ (μg/m^3^)	References
Acrylonitrile	-	6.80 × 10^−5^	2.38 × 10^−4^	2.00 × 10^−3^	5.71 × 10^−1^	-	[23,24]
Methylene Chloride	2.08 × 10^3^	1.00 × 10^−8^	3.50 × 10^−8^	6.00 × 10^−1^	1.71 × 10^2^	-	[19,25,26]
1,2-Dichloroethane	4.05 × 10^2^	2.60 × 10^−5^	9.10 × 10^−5^	-	-	-	[19,27]
Benzene	2.88 × 10^1^	7.80 × 10^−5^	2.73 × 10^−5^	3.00 × 10^−2^	8.57 × 10^0^	30	[16,19,28,29]
Toluene	7.54 × 10^3^	-	-	5.00 × 10^0^	1.43 × 10^3^	1000	[16,19,30]
Ethylbenzene	2.17 × 10^4^	-	-	1.00 × 10^0^	2.86 × 10^2^	1000	[16,19,31]
m-Xylene	8.68 × 10^3^	-	-	1.00 × 10^−1^	2.86 × 10^1^	870	[16,19,32]
p-Xylene	8.68 × 10^3^	-	-	1.00 × 10^−1^	2.86 × 10^1^	870	[16,19,32]
Styrene	2.13 × 10^4^	-	-	1.00 × 10^0^	2.86 × 10^2^	220	[16,19,33]
o-Xylene	8.68 × 10^3^	-	-	1.00 × 10^−1^	2.86 × 10^1^	870	[16,19,32]
1,2,4-Trimethylbenzene	-	-	-	6.00 × 10^−2^	1.71 × 10^1^	-	[34]
Methyl Ethyl Ketone	2.95 × 10^3^	-	-	5.00 × 10^0^	1.43 × 10^3^	-	[19,35]
Hexane	2.11 × 10^4^	-	-	7.00 × 10^−1^	2.00 × 10^2^	-	[19,36]
Formaldehyde	4.91 × 10^1^	1.10 × 10^−5^	3.85 × 10^−5^	7.00 × 10^−3^	2.00 × 10^0^	210	[16,19,37,38]
Acetaldehyde	-	2.20 × 10^−5^	7.70 × 10^−5^	9.00 × 10^−3^	2.57 × 10^0^	300	[16,39,40]
Acrolein	6.88 × 10^0^	-	-	2.00 × 10^−5^	5.71 × 10^−3^	50	[16,19,41]
Propionaldehyde	-	-	-	8.00 × 10^−3^	2.29 × 10^0^	-	[42]
Ammonia	1.18 × 10^3^	-	-	5.00 × 10^−1^	1.43 × 10^2^	-	[19,43]

^a^ MRL: Minimal Risk Level; ^b^ UR: Unit Risk; ^c^ CPF: Cancer Potency Factor; ^d^ RfC: Reference Concentration; ^e^ RfD: Reference Dose; ^f^ Standard value specified in the “Indoor Air Quality Guidelines for Newly Manufactured Automobile” of the MOLIT.

**Table 4:** Concentration of pollutants by test mode for each automobile (unit: μg/m^3^).

Pollutants	Automobile A					Automobile B					Automobile C				
	AM ^a^	PM ^b^ (2 h)	PM ^b^ (4 h)	PM ^b^ (6 h)	DM ^c^	AM	PM (2 h)	PM (4 h)	PM (6 h)	DM	AM	PM (2 h)	PM (4 h)	PM (6 h)	DM
Acrylonitrile	9.1	15.7	18.5	19.1	4.3	6.3	16.8	21.8	25.5	13.4	N.D. ^d^	5.8	5.9	5.9	N.D.
Methylene Chloride	9.3	12.6	13.1	13.8	9.6	N.D.	8.6	9.5	10.9	7.9	N.D.	6.8	N.D.	N.D.	N.D.
1,2-Dichloroethane	N.D.	N.D.	13.2	14.3	N.D.	N.D.	N.D.	N.D.	N.D.	N.D.	N.D.	N.D.	N.D.	N.D.	N.D.
Benzene	5.2	6.5	6.9	7.1	N.D.	7.7	13.8	15.2	16.9	6.0	3.7	4.4	4.1	3.9	N.D.
Toluene	119.0	218.2	249.2	251.3	31.6	130.6	172.8	209.5	229.4	79.3	29.3	32.6	32.9	32.6	6.0
Ethylbenzene	36.3	51.7	54.9	54.2	10.8	81.6	103.1	124.6	132.1	53.3	62.6	66.2	61.4	56.1	10.1
m-Xylene	27.8	48.1	53.3	57.1	9.9	58.3	78.2	95.1	107.4	42.8	22.2	24.8	22.7	21.8	N.D.
p-Xylene	10.7	17.4	21.0	21.4	N.D.	15.2	20.3	28.9	25.1	11.1	N.D.	9.8	9.7	N.D.	N.D.
Styrene	7.8	17.5	22.1	24.0	3.7	12.0	17.3	21.5	24.2	9.7	4.0	5.2	5.5	5.7	N.D.
o-Xylene	18.8	35.1	42.2	44.5	7.1	40.9	55.4	71.2	80.5	31.2	11.1	12.7	12.5	12.0	N.D.
1,2,4-Trimethylbenzene	22.4	56.6	72.3	79.9	16.2	23.8	35.2	44.8	51.2	24.5	6.8	9.4	10.3	10.9	2.7
Methyl Ethyl Ketone	52.8	99.9	112.7	182.2	110.0	60.7	83.1	105.9	119.8	41.9	17.0	22.7	26.4	27.1	N.D.
Hexane	23.0	29.8	37.2	45.5	N.D.	32.1	42.7	67.9	94.2	31.7	10.0	10.5	N.D.	13.2	N.D.
Formaldehyde	14.0	42.6	46.5	49.0	10.0	24.1	64.0	80.3	84.7	16.7	14.8	30.9	35.6	37.4	6.5
Acetaldehyde	48.0	141.0	147.4	161.4	10.3	69.1	97.1	119.8	131.6	48.9	24.2	33.7	38.2	39.2	12.0
Acrolein	N.D.	2.9	3.5	4.6	N.D.	2.7	3.6	4.5	5.1	2.2	2.1	2.5	2.7	3.2	N.D.
Propionaldehyde	27.6	63.0	86.0	100.6	2.8	17.4	27.0	34.0	37.5	7.9	4.2	6.0	6.5	7.2	N.D.
Ammonia	125.4	242.2	320.7	361.6	130.3	122.6	406.1	464.0	537.1	120.3	24.6	57.8	64.7	66.7	30.7
**Pollutants**		**Automobile D**							**Automobile E**						
		**AM ^a^**	**PM ^b^ (2 h)**		**PM ^b^ (4 h)**		**PM ^b^ (6 h)**	**DM ^c^**	**AM**	**PM (2 h)**		**PM (4 h)**		**PM (6 h)**	**DM**
Acrylonitrile		5.1	15.0		15.0		26.2	10.9	N.D. ^d^	N.D.		21.7		4.0	N.D.
Methylene Chloride		N.D.	10.3		10.3		13.2	7.8	N.D.	N.D.		11.6		N.D.	N.D.
1,2-Dichloroethane		N.D.	N.D.		N.D.		13.2	N.D.	N.D.	21.7		N.D.		79.4	17.6
Benzene		2.9	4.2		4.2		5.6	N.D.	N.D.	2.5		4.9		6.6	2.6
Toluene		37.1	56.8		56.8		84.5	17.2	14.4	37.4		73.8		87.3	33.7
Ethylbenzene		39.6	56.6		56.6		74.8	17.9	34.3	100.7		68.4		286.3	113.6
m-Xylene		22.4	35.8		35.8		53.6	12.4	60.6	264.7		46.7		943.6	382.0
p-Xylene		N.D.	12.3		12.3		17.6	N.D.	19.6	68.7		15.3		251.7	101.1
Styrene		4.7	11.1		11.1		19.4	4.8	N.D.	9.9		16.0		41.5	22.3
o-Xylene		16.8	28.2		28.2		45.8	10.5	48.6	216.3		38.8		795.2	349.5
1,2,4-Trimethylbenzene		16.5	33.7		33.7		52.8	13.9	61.2	261.1		46.5		884.8	502.7
Methyl Ethyl Ketone		41.4	62.3		62.3		94.2	32.0	7.7	18.6		81.8		23.9	11.6
Hexane		14.7	28.3		28.3		42.5	11.7	N.D.	N.D.		36.7		35.9	9.9
Formaldehyde		25.5	70.0		70.0		87.6	19.7	13.2	55.5		84.4		129.0	71.5
Acetaldehyde		25.0	45.0		45.0		76.5	22.1	11.1	22.5		60.5		83.6	15.1
Acrolein		N.D.	4.6		4.6		5.5	N.D.	N.D.	1.8		5.1		9.1	N.D.
Propionaldehyde		6.3	10.2		10.2		17.6	2.3	2.6	2.6		6.3		13.9	26.1
Ammonia		186.6	822.9		822.9		867.2	177.4	51.4	1591.6		883.5		2494.6	73.6

^a^ AM: Ambient Mode; ^b^ PM: Parking Mode; ^c^ DM: Driving Mode; ^d^ N.D.: Not Detected.

**Table 5:** Automobile indoor air quality (IAQ) grading.

Automobile IAQ Grading			Acute Risk (HQ_acute_ ^a^)	Chronic Risk	
				ECR ^b^	HQ ^c^
Grade 1	Excellent	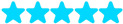	<1	<10^−6^	<1.0
Grade 2	Very Good	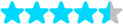	<1	<10^−6^	>1.0
Grade 3	Good	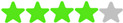	<1	>10^−6^	<0.1
Grade 4	Moderate (High)	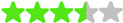	<1	>10^−5^	>1.0
Grade 5	Moderate	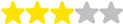	<1	<10^−5^	>0.1
Grade 6	Moderate (Low)	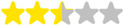	<1	>10^−5^	<0.1
Grade 7	Slightly Unhealthy	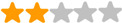	<1	>10^−5^	>0.1
Grade 8	Unhealthy	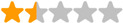	>1	<10^−4^	<1.0
Grade 9	Very Unhealthy	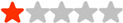	>1	<10^−4^	>0.1
Grade 10	Hazardous	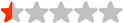	>1	>10^−4^	>0.1

^a^ HQ_acute_: Acute Hazard Quotient; ^b^ ECR: Excess Cancer Risk; ^c^ HQ: Hazard Quotient.

**Table 6:** Results of acute and chronic risk assessment for Automobile A.

Pollutants	AM			PM (2 h)			PM (4 h)			PM (6 h)			DM		
	HQ_acute_	HQ	ECR	HQ_acute_	HQ	ECR	HQ_acute_	HQ	ECR	HQ_acute_	HQ	ECR	HQ_acute_	HQ	ECR
Acrylonitrile	-	2.29 × 10^−1^	5.65 × 10^−6^	-	3.95 × 10^−1^	9.74 × 10^−6^	-	4.65 × 10^−1^	1.15 × 10^−5^	-	4.81 × 10^−1^	1.19 × 10^−5^	-	1.08 × 10^−1^	2.67 × 10^−6^
Methylene Chloride	4.46 × 10^−3^	7.80 × 10^−4^	8.49 × 10^−10^	6.05 × 10^−3^	1.06 × 10^−3^	1.15 × 10^−9^	6.29 × 10^−3^	1.10 × 10^−3^	1.20 × 10^−9^	6.62 × 10^−3^	1.16 × 10^−3^	1.26 × 10^−9^	4.61 × 10^−3^	8.05 × 10^−4^	8.76 × 10^−10^
1,2-Dichloroethane	-	-	-	-	-	-	-	-	-	-	-	-	-	-	-
Benzene	1.81 × 10^−1^	8.72 × 10^−3^	3.70 × 10^−7^	2.26 × 10^−1^	1.09 × 10^−2^	4.63 × 10^−7^	2.40 × 10^−1^	1.16 × 10^−2^	4.91 × 10^−7^	2.47 × 10^−1^	1.19 × 10^−2^	5.05 × 10^−7^	-	-	-
Toluene	1.58 × 10^−2^	1.20 × 10^−3^	-	2.90 × 10^−2^	2.20 × 10^−3^	-	3.31 × 10^−2^	2.51 × 10^−3^	-	3.33 × 10^−2^	2.53 × 10^−3^	-	4.19 × 10^−3^	3.18 × 10^−4^	-
Ethylbenzene	1.67 × 10^−3^	1.83 × 10^−3^	-	2.38 × 10^−3^	2.60 × 10^−3^	-	2.53 × 10^−3^	2.76 × 10^−3^	-	2.50 × 10^−3^	2.73 × 10^−3^	-	4.97 × 10^−4^	5.43 × 10^−4^	-
m-Xylene	3.20 × 10^−3^	1.40 × 10^−2^	-	5.54 × 10^−3^	2.42 × 10^−2^	-	6.14 × 10^−3^	2.68 × 10^−2^	-	6.58 × 10^−3^	2.87 × 10^−2^	-	1.14 × 10^−3^	4.98 × 10^−3^	-
p-Xylene	1.23 × 10^−3^	5.38 × 10^−3^	-	2.00 × 10^−3^	8.76 × 10^−3^	-	2.42 × 10^−3^	1.06 × 10^−2^	-	2.46 × 10^−3^	1.08 × 10^−2^	-	-	-	-
Styrene	3.66 × 10^−4^	3.92 × 10^−4^	-	8.22 × 10^−4^	8.81 × 10^−4^	-	1.04 × 10^−3^	1.11 × 10^−3^	-	1.13 × 10^−3^	1.21 × 10^−3^	-	1.74 × 10^−4^	1.86 × 10^−4^	-
o-Xylene	2.16 × 10^−3^	9.46 × 10^−3^	-	4.04 × 10^−3^	1.77 × 10^−2^	-	4.86 × 10^−3^	2.12 × 10^−2^	-	4.86 × 10^−3^	2.24 × 10^−2^	-	8.18 × 10^−4^	3.57 × 10^−3^	-
1,2,4-Trimethylbenzene	-	1.88 × 10^−2^	-	-	4.75 × 10^−2^	-	-	6.06 × 10^−2^	-	-	6.70 × 10^−2^	-	-	1.36 × 10^−2^	-
Methyl Ethyl Ketone	1.79 × 10^−2^	5.31 × 10^−4^	-	3.39 × 10^−2^	1.01 × 10^−3^	-	3.82 × 10^−2^	1.13 × 10^−3^	-	3.82 × 10^−2^	1.83 × 10^−3^	-	3.73 × 10^−2^	1.11 × 10^−3^	-
Hexane	1.09 × 10^−3^	1.65 × 10^−3^	-	1.41 × 10^−3^	2.14 × 10^−3^	-	1.76 × 10^−3^	2.67 × 10^−3^	-	1.76 × 10^−3^	3.27 × 10^−3^	-	-	-	-
Formaldehyde	2.85 × 10^−1^	1.01 × 10^−1^	1.41 × 10^−6^	8.67 × 10^−1^	3.06 × 10^−1^	4.28 × 10^−6^	9.47 × 10^−1^	3.34 × 10^−1^	4.67 × 10^−6^	9.47 × 10^−1^	352 × 10^−1^	4.92 × 10^−6^	2.04 × 10^−1^	7.19 × 10^−2^	1.00 × 10^−6^
Acetaldehyde	-	2.68 × 10^−1^	9.64 × 10^−7^	-	7.88 × 10^−1^	2.83 × 10^−6^	-	8.24 × 10^−1^	2.96 × 10^−6^	-	9.02 × 10^−1^	3.24 × 10^−6^	-	5.76 × 10^−2^	2.07 × 10^−7^
Acrolein	-	-	-	4.22 × 10^−1^	7.30 × 10^0^	-	5.09 × 10^−1^	8.81 × 10^0^	-	5.09 × 10^−1^	1.16 × 10^1^	-	-	-	-
Propionaldehyde	-	1.74 × 10^−1^	-	-	3.96 × 10^−1^	-	-	5.41 × 10^−1^	-	-	6.33 × 10^−1^	-	-	1.76 × 10^−2^	-
Ammonia	1.06 × 10^−1^	1.26 × 10^−2^	-	2.05 × 10^−1^	2.44 × 10^−2^	-	2.71 × 10^−1^	3.23 × 10^−2^	-	2.71 × 10^−1^	3.64 × 10^−2^	-	1.10 × 10^−1^	1.31 × 10^−2^	-

**Table 7:** Results of acute and chronic risk assessment for Automobile B.

Pollutants	AM			PM (2 h)			PM (4 h)			PM (6 h)			DM		
	HQ_acute_	HQ	ECR	HQ_acute_	HQ	ECR	HQ_acute_	HQ	ECR	HQ_acute_	HQ	ECR	HQ_acute_	HQ	ECR
Acrylonitrile	-	1.59 × 10^−1^	3.91 × 10^−6^	-	4.23 × 10^−1^	1.04 × 10^−5^	-	5.48 × 10^−1^	1.35 × 10^−5^	-	6.42 × 10^−1^	1.58 × 10^−5^	-	3.37 × 10^−1^	8.32 × 10^−6^
Methylene Chloride	-	-	-	4.13 × 10^−3^	7.21 × 10^−4^	7.85 × 10^−10^	4.56 × 10^−3^	7.97 × 10^−4^	8.67 × 10^−10^	5.23 × 10^−3^	9.14 × 10^−4^	9.95 × 10^−10^	3.79 × 10^−3^	6.63 × 10^−4^	7.21 × 10^−10^
1,2-Dichloroethane	-	-	-	-	-	-	-	-	-	-	-	-	-	-	-
Benzene	2.68 × 10^−1^	1.29 × 10^−2^	5.48 × 10^−7^	4.80 × 10^−1^	2.31 × 10^−2^	9.82 × 10^−7^	5.29 × 10^−1^	2.55 × 10^−2^	1.08 × 10^−6^	5.88 × 10^−1^	2.83 × 10^−2^	1.20 × 10^−6^	2.09 × 10^−1^	1.01 × 10^−2^	4.27 × 10^−7^
Toluene	1.73 × 10^−2^	1.31 × 10^−3^	-	2.29 × 10^−2^	1.74 × 10^−3^	-	2.78 × 10^−2^	2.11 × 10^−3^	-	3.04 × 10^−2^	2.31 × 10^−3^	-	1.05 × 10^−2^	7.98 × 10^−4^	-
Ethylbenzene	3.76 × 10^−3^	4.11 × 10^−3^	-	4.75 × 10^−3^	5.19 × 10^−3^	-	5.74 × 10^−3^	6.27 × 10^−3^	-	6.08 × 10^−3^	6.65 × 10^−3^	-	2.46 × 10^−3^	2.68 × 10^−3^	-
m-Xylene	6.71 × 10^−3^	2.93 × 10^−2^	-	9.01 × 10^−3^	3.93 × 10^−2^	-	1.10 × 10^−2^	4.79 × 10^−2^	-	1.24 × 10^−2^	5.40 × 10^−2^	-	4.93 × 10^−3^	2.15 × 10^−2^	-
p-Xylene	1.75 × 10^−3^	7.65 × 10^−3^	-	2.34 × 10^−3^	1.02 × 10^−2^	-	3.33 × 10^−3^	1.45 × 10^−2^	-	2.89 × 10^−3^	1.26 × 10^−2^	-	1.28 × 10^−3^	5.59 × 10^−3^	-
Styrene	5.63 × 10^−4^	6.04 × 10^−4^	-	8.12 × 10^−4^	8.70 × 10^−4^	-	1.01 × 10^−3^	1.08 × 10^−3^	-	1.14 × 10^−3^	1.22 × 10^−3^	-	-	4.88 × 10^−4^	-
o-Xylene	4.71 × 10^−3^	2.06 × 10^−2^	-	6.38 × 10^−3^	2.79 × 10^−2^	-	8.20 × 10^−3^	3.58 × 10^−2^	-	9.27 × 10^−3^	4.05 × 10^−2^	-	3.59 × 10^−3^	1.57 × 10^−2^	-
1,2,4-Trimethylbenzene	-	2.00 × 10^−2^	-	-	2.95 × 10^−2^	-	-	3.76 × 10^−2^	-	-	4.29 × 10^−2^	-	-	2.05 × 10^−2^	-
Methyl Ethyl Ketone	2.06 × 10^−2^	6.11 × 10^−4^	-	2.82 × 10^−2^	8.35 × 10^−4^	-	3.59 × 10^−2^	1.07 × 10^−3^	-	4.06 × 10^−2^	1.21 × 10^−3^	-	1.42 × 10^−2^	4.22 × 10^−4^	-
Hexane	1.52 × 10^−3^	2.31 × 10^−3^	-	2.02 × 10^−3^	3.07 × 10^−3^	-	3.21 × 10^−3^	4.88 × 10^−3^	-	4.45 × 10^−3^	6.77 × 10^−3^	-	1.50 × 10^−3^	2.28 × 10^−3^	-
Formaldehyde	4.91 × 10^−1^	1.73 × 10^−1^	2.42 × 10^−6^	1.30 × 10^0^	4.60 × 10^−1^	6.43 × 10^−6^	1.63 × 10^0^	5.77 × 10^−1^	8.06 × 10^−6^	1.72 × 10^0^	6.09 × 10^−1^	8.50 × 10^−6^	3.40 × 10^−1^	1.20 × 10^−1^	1.68 × 10^−6^
Acetaldehyde	-	3.86 × 10^−1^	1.39 × 10^−6^	-	5.43 × 10^−1^	1.95 × 10^−6^	-	6.70 × 10^−1^	2.41 × 10^−6^	-	7.36 × 10^−1^	2.64 × 10^−6^	-	2.73 × 10^−1^	9.82 × 10^−7^
Acrolein	3.93 × 10^−1^	6.79 × 10^0^	-	5.23 × 10^−1^	9.06 × 10^0^	-	6.54 × 10^−1^	1.13 × 10^1^	-	7.41 × 10^−1^	1.28 × 10^1^	-	3.20 × 10^−1^	5.53 × 10^0^	-
Propionaldehyde	-	1.09 × 10^−1^	-	-	1.70 × 10^−1^	-	-	2.14 × 10^−1^	-	-	2.36 × 10^−1^	-	-	4.97 × 10^−2^	-
Ammonia	1.04 × 10^−1^	1.23 × 10^−2^	-	3.43 × 10^−1^	4.09 × 10^−2^	-	3.92 × 10^−1^	4.67 × 10^−2^	-	4.54 × 10^−1^	5.41 × 10^−2^	-	1.02 × 10^−1^	1.21 × 10^−2^	-

**Table 8:** Results of acute and chronic risk assessment for Automobile C.

Pollutants	AM			PM (2 h)			PM (4 h)			PM (6 h)			DM		
	HQ_acute_	HQ	ECR	HQ_acute_	HQ	ECR	HQ_acute_	HQ	ECR	HQ_acute_	HQ	ECR	HQ_acute_	HQ	ECR
Acrylonitrile	-	-	-	-	1.46 × 10^−1^	3.60 × 10^−6^	-	1.48 × 10^−1^	3.66 × 10^−6^	-	1.48 × 10^−1^	3.66 × 10^−6^	-	-	-
Methylene Chloride	-	-	-	3.23 × 10^−3^	5.70 × 10^−4^	6.21 × 10^−10^	-	-	-	-	-	-	-	-	-
1,2-Dichloroethane	-	-	-	-	-	-	-	-	-	-	-	-	-	-	-
Benzene	1.29 × 10^−1^	6.21 × 10^−3^	2.63 × 10^−7^	1.53 × 10^−1^	7.38 × 10^−3^	3.13 × 10^−7^	1.43 × 10^−1^	6.88 × 10^−3^	2.92 × 10^−7^	1.36 × 10^−1^	6.54 × 10^−3^	2.78 × 10^−7^	-	-	-
Toluene	3.89 × 10^−3^	2.95 × 10^−4^	-	4.33 × 10^−3^	3.28 × 10^−4^	-	4.37 × 10^−3^	3.31 × 10^−4^	-	4.33 × 10^−3^	3.28 × 10^−4^	-	7.96 × 10^−4^	6.04 × 10^−5^	-
Ethylbenzene	2.88 × 10^−3^	3.15 × 10^−3^	-	3.05 × 10^−3^	3.33 × 10^−3^	-	2.83 × 10^−3^	3.09 × 10^−3^	-	2.58 × 10^−3^	2.82 × 10^−3^	-	4.65 × 10^−4^	5.08 × 10^−4^	-
m-Xylene	2.56 × 10^−2^	1.12 × 10^−2^	-	2.86 × 10^−3^	1.25 × 10^−2^	-	2.61 × 10^−3^	1.14 × 10^−2^	-	2.51 × 10^−3^	1.10 × 10^−2^	-	-	-	-
p-Xylene	-	-	-	1.13 × 10^−3^	4.93 × 10^−3^	-	1.12 × 10^−3^	4.88 × 10^−3^	-	-	-	-	-	-	-
Styrene	1.88 × 10^−4^	2.01 × 10^−4^	-	2.44 × 10^−4^	2.62 × 10^−4^	-	2.58 × 10^−4^	2.77 × 10^−4^	-	2.68 × 10^−4^	2.87 × 10^−4^	-	-	-	-
o-Xylene	1.28 × 10^−3^	5.59 × 10^−3^	-	1.46 × 10^−3^	6.39 × 10^−3^	-	1.44 × 10^−3^	6.29 × 10^−3^	-	1.38 × 10^−3^	6.04 × 10^−3^	-	-	-	-
1,2,4-Trimethylbenzene	-	5.70 × 10^−3^	-	-	7.88 × 10^−3^	-	-	8.64 × 10^−3^	-	-	9.14 × 10^−3^	-	-	2.26 × 10^−3^	-
Methyl Ethyl Ketone	5.76 × 10^−3^	1.71 × 10^−4^	-	7.70 × 10^−3^	2.28 × 10^−4^	-	8.95 × 10^−3^	2.66 × 10^−4^	-	9.19 × 10^−3^	2.73 × 10^−4^	-	-	-	-
Hexane	4.73 × 10^−4^	7.19 × 10^−4^	-	4.96 × 10^−4^	7.55 × 10^−4^	-	-	-	-	6.24 × 10^−4^	9.49 × 10^−4^	-	-	-	-
Formaldehyde	3.01 × 10^−1^	1.06 × 10^−1^	1.49 × 10^−6^	6.29 × 10^−1^	2.22 × 10^−1^	3.10 × 10^−6^	7.25 × 10^−1^	2.56 × 10^−1^	3.57 × 10^−6^	7.61 × 10^−1^	2.69 × 10^−1^	3.75 × 10^−6^	1.32 × 10^−1^	4.67 × 10^−2^	6.53 × 10^−7^
Acetaldehyde	-	1.35 × 10^−1^	4.86 × 10^−7^	-	1.88 × 10^−1^	6.77 × 10^−7^	-	2.14 × 10^−1^	7.67 × 10^−7^	-	2.19 × 10^−1^	7.87 × 10^−7^	-	6.71 × 10^−2^	2.41 × 10^−7^
Acrolein	3.05 × 10^−1^	5.28 × 10^0^	-	3.63 × 10^−1^	6.29 × 10^0^	-	3.93 × 10^−1^	6.79 × 10^0^	-	4.65 × 10^−1^	8.05 × 10^0^	-	-	-	-
Propionaldehyde	-	2.64 × 10^−2^	-	-	3.77 × 10^−2^	-	-	4.09 × 10^−2^	-	-	4.53 × 10^−2^	-	-	-	-
Ammonia	2.08 × 10^−2^	2.48 × 10^−3^	-	4.88 × 10^−2^	5.82 × 10^−3^	-	5.46 × 10^−2^	6.51 × 10^−3^	-	5.63 × 10^−2^	6.71 × 10^−3^	-	2.59 × 10^−2^	3.09 × 10^−3^	-

**Table 9:** Results of acute and chronic risk assessment for Automobile D.

Pollutants	AM			PM (2 h)			PM (4 h)			PM (6 h)			DM		
	HQ_acute_	HQ	ECR	HQ_acute_	HQ	ECR	HQ_acute_	HQ	ECR	HQ_acute_	HQ	ECR	HQ_acute_	HQ	ECR
Acrylonitrile	-	1.28 × 10^−1^	3.17 × 10^−6^	-	3.77 × 10^−1^	9.31 × 10^−6^	-	3.77 × 10^−1^	9.31 × 10^−6^	-	6.59 × 10^−1^	1.63 × 10^−5^	-	2.74 × 10^−1^	6.76 × 10^−6^
Methylene Chloride	-	-	-	4.94 × 10^−3^	8.64 × 10^−4^	9.40 × 10^−10^	4.94 × 10^−3^	8.64 × 10^−4^	9.40 × 10^−10^	6.33 × 10^−3^	1.11 × 10^−3^	1.20 × 10^−9^	3.74 × 10^−3^	6.54 × 10^−4^	7.12 × 10^−10^
1,2-Dichloroethane	-	-	-	-	-	-	-	-	-	3.26 × 10^−2^	-	-	-	-	-
Benzene	1.01 × 10^−1^	4.86 × 10^−3^	2.06 × 10^−7^	1.46 × 10^−1^	7.04 × 10^−3^	2.99 × 10^−7^	1.46 × 10^−1^	7.04 × 10^−3^	2.99 × 10^−7^	1.95 × 10^−1^	9.39 × 10^−3^	3.99 × 10^−7^	-	-	-
Toluene	4.92 × 10^−3^	3.73 × 10^−4^	-	7.54 × 10^−3^	5.72 × 10^−4^	-	7.54 × 10^−3^	5.72 × 10^−4^	-	1.12 × 10^−2^	8.50 × 10^−4^	-	2.28 × 10^−3^	1.73 × 10^−4^	-
Ethylbenzene	1.82 × 10^−3^	1.99 × 10^−3^	-	2.61 × 10^−3^	2.85 × 10^−3^	-	2.61 × 10^−3^	2.85 × 10^−3^	-	3.45 × 10^−3^	3.76 × 10^−3^	-	8.25 × 10^−4^	9.01 × 10^−4^	-
m-Xylene	2.58 × 10^−3^	1.13 × 10^−2^	-	4.12 × 10^−3^	1.80 × 10^−2^	-	4.12 × 10^−3^	1.80 × 10^−2^	-	6.17 × 10^−3^	2.70 × 10^−2^	-	1.43 × 10^−3^	6.24 × 10^−3^	-
p-Xylene	-	-	-	1.42 × 10^−3^	6.19 × 10^−3^	-	1.42 × 10^−3^	6.19 × 10^−3^	-	2.03 × 10^−3^	8.86 × 10^−3^	-	-	-	-
Styrene	2.21 × 10^−4^	2.36 × 10^−4^	-	5.21 × 10^−4^	5.59 × 10^−4^	-	5.21 × 10^−4^	5.59 × 10^−4^	-	9.11 × 10^−4^	9.76 × 10^−4^	-	2.25 × 10^−4^	2.42 × 10^−4^	-
o-Xylene	1.93 × 10^−3^	8.45 × 10^−3^	-	3.25 × 10^−3^	1.42 × 10^−2^	-	3.25 × 10^−3^	1.42 × 10^−2^	-	5.27 × 10^−3^	2.30 × 10^−2^	-	1.21 × 10^−3^	5.28 × 10^−3^	*
1,2,4-Trimethylbenzene	-	1.38 × 10^−2^	-	-	2.83 × 10^−2^	-	-	2.83 × 10^−2^	-	-	4.43 × 10^−2^	-	-	1.17 × 10^−2^	-
Methyl Ethyl Ketone	1.40 × 10^−2^	4.17 × 10^−4^	-	2.11 × 10^−2^	6.27 × 10^−4^	-	2.11 × 10^−2^	6.27 × 10^−4^	-	3.19 × 10^−2^	9.48 × 10^−4^	-	1.09 × 10^−2^	3.22 × 10^−4^	-
Hexane	6.95 × 10^−4^	1.06 × 10^−3^	-	1.34 × 10^−3^	2.03 × 10^−3^	-	1.34 × 10^−3^	2.03 × 10^−3^	-	2.01 × 10^−3^	3.06 × 10^−3^	-	5.53 × 10^−4^	8.41 × 10^−4^	-
Formaldehyde	5.19 × 10^−1^	1.83 × 10^−1^	2.56 × 10^−6^	1.43 × 10^0^	5.03 × 10^−1^	7.03 × 10^−6^	1.43 × 10^0^	5.03 × 10^−1^	7.03 × 10^−6^	1.78 × 10^0^	6.30 × 10^−1^	8.79 × 10^−6^	4.01 × 10^−1^	1.42 × 10^−1^	1.98 × 10^−6^
Acetaldehyde	-	1.40 × 10^−1^	5.02 × 10^−7^	-	2.52 × 10^−1^	9.04 × 10^−7^	-	2.52 × 10^−1^	9.04 × 10^−7^	-	4.28 × 10^−1^	1.54 × 10^−6^	-	1.24 × 10^−1^	4.44 × 10^−7^
Acrolein	-	-	-	6.69 × 10^−1^	1.16 × 10^1^	-	6.69 × 10^−1^	1.16 × 10^1^	-	8.00 × 10^−1^	1.38 × 10^1^	-	-	-	-
Propionaldehyde	-	3.96 × 10^−2^	-	-	6.42 × 10^−2^	-	-	6.42 × 10^−2^	-	-	1.11 × 10^−1^	-	-	1.45 × 10^−2^	-
Ammonia	1.58 × 10^−1^	1.88 × 10^−2^	-	6.95 × 10^−1^	8.28 × 10^−2^	-	6.95 × 10^−1^	8.28 × 10^−2^	-	7.32 × 10^−1^	8.73 × 10^−2^	-	1.50 × 10^−1^	1.79 × 10^−2^	-

**Table 10:** Results of acute and chronic risk assessment for Automobile E.

Pollutants	AM			PM (2 h)			PM (4 h)			PM (6 h)			DM		
	HQ_acute_	HQ	ECR	HQ_acute_	HQ	ECR	HQ_acute_	HQ	ECR	HQ_acute_	HQ	ECR	HQ_acute_	HQ	ECR
Acrylonitrile	-	-	-	-	-	-	-	5.46 × 10^−1^	1.35 × 10^−5^	-	1.01 × 10^−1^	2.48 × 10^−6^	-	-	-
Methylene Chloride	-	-	-	-	-	-	5.57 × 10^−3^	9.73 × 10^−4^	1.06 × 10^−9^	-	-	-	-	-	-
1,2-Dichloroethane	-	-	-	5.36 × 10^−2^	-	-	-	-	-	1.96 × 10^−1^	-	-	4.35 × 10^−2^	-	-
Benzene	-	-	-	8.70 × 10^−2^	4.19 × 10^−3^	1.78 × 10^−7^	1.70 × 10^−1^	8.22 × 10^−3^	3.49 × 10^−7^	2.30 × 10^−1^	1.11 × 10^−2^	4.70 × 10^−7^	9.04 × 10^−2^	4.36 × 10^−3^	1.85 × 10^−7^
Toluene	1.91 × 10^−3^	1.45 × 10^−4^	-	4.96 × 10^−3^	3.76 × 10^−4^	-	9.79 × 10^−3^	7.43 × 10^−4^	-	1.16 × 10^−2^	8.79 × 10^−4^	-	4.47 × 10^−3^	3.39 × 10^−4^	-
Ethylbenzene	1.58 × 10^−3^	1.73 × 10^−3^	-	4.64 × 10^−3^	5.07 × 10^−3^	-	3.15 × 10^−3^	3.44 × 10^−3^	-	1.32 × 10^−2^	1.44 × 10^−2^	-	5.23 × 10^−3^	5.72 × 10^−3^	-
m-Xylene	6.98 × 10^−3^	3.05 × 10^−2^	-	3.05 × 10^−2^	1.33 × 10^−1^	-	5.38 × 10^−3^	2.35 × 10^−2^	-	1.09 × 10^−1^	4.75 × 10^−1^	-	4.40 × 10^−2^	1.92 × 10^−1^	-
p-Xylene	2.26 × 10^−3^	9.86 × 10^−3^	-	7.91 × 10^−3^	3.46 × 10^−2^	-	1.76 × 10^−3^	7.70 × 10^−3^	-	2.90 × 10^−2^	1.27 × 10^−1^	-	1.16 × 10^−2^	5.09 × 10^−2^	-
Styrene	-	-	-	4.65 × 10^−4^	4.98 × 10^−4^	-	7.51 × 10^−4^	8.05 × 10^−4^	-	1.95 × 10^−3^	2.09 × 10^−3^	-	1.05 × 10^−3^	1.12 × 10^−3^	-
o-Xylene	5.60 × 10^−3^	2.45 × 10^−2^	-	2.49 × 10^−2^	1.09 × 10^−1^	-	4.47 × 10^−3^	1.95 × 10^−2^	-	9.16 × 10^−2^	4.00 × 10^−1^	-	4.02 × 10^−2^	1.76 × 10^−1^	-
1,2,4-Trimethylbenzene	-	5.13 × 10^−2^	-	-	2.19 × 10^−1^	-	-	3.90 × 10^−2^	-	-	7.42 × 10^−1^	-	-	4.22 × 10^−1^	-
Methyl Ethyl Ketone	2.61 × 10^−3^	7.75 × 10^−5^	-	6.31 × 10^−3^	1.87 × 10^−4^	-	2.77 × 10^−2^	8.23 × 10^−4^	-	8.10 × 10^−3^	2.41 × 10^−4^	-	3.93 × 10^−3^	1.17 × 10^−4^	-
Hexane	-	-	-	-	-	-	1.74 × 10^−3^	2.64 × 10^−3^	-	1.70 × 10^−3^	2.58 × 10^−3^	-	4.68 × 10^−4^	7.12 × 10^−4^	-
Formaldehyde	2.69 × 10^−1^	9.49 × 10^−2^	1.33 × 10^−6^	1.13 × 10^0^	3.99 × 10^−1^	5.57 × 10^−6^	1.72 × 10^0^	6.07 × 10^−1^	8.47 × 10^−6^	2.63 × 10^0^	9.27 × 10^−1^	1.30 × 10^−6^	1.46 × 10^0^	5.14 × 10^−1^	7.18 × 10^−6^
Acetaldehyde	-	6.21 × 10^−2^	2.23 × 10^−7^	-	1.26 × 10^−1^	4.52 × 10^−7^	-	3.38 × 10^−1^	1.21 × 10^−6^	-	4.67 × 10^−1^	1.68 × 10^−6^	-	8.44 × 10^−2^	3.03 × 10^−7^
Acrolein	-	-	-	2.62 × 10^−1^	4.53 × 10^0^	-	7.41 × 10^−1^	1.28 × 10^1^	-	1.32 × 10^0^	2.29 × 10^1^	-	-	-	-
Propionaldehyde	-	1.64 × 10^−2^	-	-	3.96 × 10^−2^	-	-	8.74 × 10^−2^	-	-	1.64 × 10^−1^	-	-	2.52 × 10^−2^	-
Ammonia	4.34 × 10^−2^	5.17 × 10^−3^	-	1.34 × 10^0^	1.60 × 10^−1^	-	7.46 × 10^−1^	8.89 × 10^−2^	-	2.11 × 10^0^	2.51 × 10^−1^	-	6.22 × 10^−2^	7.41 × 10^−3^	-

**Table 11:** The application results of automobile indoor air quality grading.

Automobile	Test Mode	Acute Risk (HQ_acute_)	Chronic Risk		Automobile IAQ Grading	
			ECR	HQ		
A	AM	<1	<10^−5^	>0.1	Grade 5	Moderate
						** 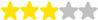 **
	PM (2 h)	<1	<10^−5^	>0.1	Grade 5	Moderate
						** 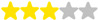 **
	PM (4 h)	<1	>10^−5^	>0.1	Grade 7	Slightly Unhealthy
						** 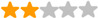 **
	PM (6 h)	<1	>10^−5^	>0.1	Grade 7	Slightly Unhealthy
						** 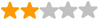 **
	DM	<1	<10^−5^	>0.1	Grade 5	Moderate
						** 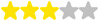 **
B	AM	<1	<10^−5^	>0.1	Grade 5	Moderate
						** 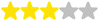 **
	PM (2 h)	>1	<10^−4^	>0.1	Grade 9	Very Unhealthy
						** 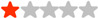 **
	PM (4 h)	>1	<10^−4^	>0.1	Grade 9	Very Unhealthy
						** 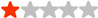 **
	PM (6 h)	>1	<10^−4^	>0.1	Grade 9	Very Unhealthy
						** 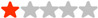 **
	DM	<1	<10^−5^	>0.1	Grade 5	Moderate
						** 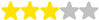 **
C	AM	<1	<10^−5^	>0.1	Grade 5	Moderate
						** 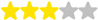 **
	PM (2 h)	<1	<10^−5^	>0.1	Grade 5	Moderate
						** 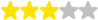 **
	PM (4 h)	<1	<10^−5^	>0.1	Grade 5	Moderate
						** 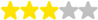 **
	PM (6 h)	<1	<10^−5^	>0.1	Grade 5	Moderate
						** 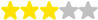 **
	DM	<1	<10^−6^	<1.0	Grade 1	Excellent
						** 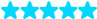 **
D	AM	<1	<10^−5^	>0.1	Grade 5	Moderate
						** 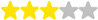 **
	PM (2 h)	>1	<10^−4^	>0.1	Grade 9	Very Unhealthy
						** 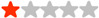 **
	PM (4 h)	>1	<10^−4^	>0.1	Grade 9	Very Unhealthy
						** 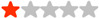 **
	PM (6 h)	>1	<10^−4^	>0.1	Grade 9	Very Unhealthy
						** 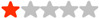 **
	DM	<1	<10^−5^	>0.1	Grade 5	Moderate
						** 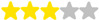 **
E	AM	<1	>10^−6^	<0.1	Grade 3	Good
						** 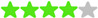 **
	PM (2 h)	>1	<10^−4^	>0.1	Grade 9	Very Unhealthy
						** 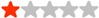 **
	PM (4 h)	>1	<10^−4^	>0.1	Grade 9	Very Unhealthy
						** 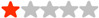 **
	PM (6 h)	The m-Xylene concentration was 943.6 μg/m^3^, exceeding the MOLIT standard of 870 μg/m^3^ and thus receiving the lowest grade (Grade 10).			Grade 10	Hazardous
						** 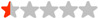 **
	DM	>1	<10^−4^	>0.1	Grade 9	Very Unhealthy
						** 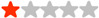 **

## Data Availability

Restrictions apply to the availability of these data. Data were obtained from MOLIT and are available with the permission of MOLIT.

## Supplementary Materials

The following supporting information can be downloaded at: https://www.mdpi.com/article/10.3390/toxics13090754/s1, Table S1: Nomenclature of abbreviations and symbols used in this study.
