# Supplementary material for: Development of an Automobile Indoor Air Quality Grading Based on Acute and Chronic Risk Assessment
Source: Toxics. 2025 Sep 4;13(9):754. doi: 10.3390/toxics13090754 (PMC12474265; doi:10.3390/toxics13090754)
Supplement: Supplementary file 1 [file toxics-13-00754-s001.zip › toxics-3824343-supplementary.pdf]

Table S1. Nomenclature of abbreviations and symbols used in this study.

Abbreviation	Definition	Unit
VOCs	Volatile Organic Compounds	-
SHS	Sick House Syndrome	-
AQI	Air Quality Index	-
API	Air Pollution Index	-
AQHI	Air Quality Health Index	-
CAI	Comprehensive Air-quality Index	-
AM	Ambient Mode	-
PM	Parking Mode	-
DM	Driving Mode	-
RH	Relative Humidity	%
GC/FID	Gas Chromatography with a Flame Ionization Detector	-
HPLC	High-Performance Liquid Chromatography	-
QA/QC	Qaulity Assurance and Quality Control	-
RT	Retention Time	-
MDL	Method Detection Limit	$\mu g/m^3$
SD	Standard Deviation	$\mu g/m^3$
HQacute	Acute Hazard Quotient	-
MRL	Minimal Risk Level	$\mu g/m^3$
ATSDR	Agency for Toxic Substances and Disease Registry	-
HRA	Health Risk Assessment	-
US EPA	United States Environmental Protection Agency	-
IRIS	Integrated Risk Information System	-
UR	Unit Risk	$(\mu g/m^3)^{-1}$
RfC	Reference Concentration	mg/m^3
CPF	Cancer Potency Factor	$(\mu g/kg/day)^{-1}$
RfD	Reference Dose	μg/kg/day
ET	Exposure Time	-
EF	Exposure Frequency	day/yr
IR	Inhalation Rate	m^3/day
BW	Body Weight	kg
ED	Exposure Duration	yr
AT	Average Time	day
LT	Lifetime	day
ECR	Excess Cancer Risk	-
LADD	Lifetime Average Daily Dose	μg/kg/day
HQ	Hazard Quotient	-
ADD	Average Daily Dose	μg/kg/day
EU	European Union	-
JAMA	Japan Automobile Manufacturers Association	-
CARB	California Air Resources Board	-
MOLIT	Ministry of Land, Infrastructure and Transport	-
